# Microcirculatory failure after successful recanalization in ischemic stroke: insights into mechanisms, imaging, and therapeutic strategies

**DOI:** 10.3389/fphar.2026.1884183

**Published:** 2026-07-20

**Authors:** Gang Wu, Lin Lin, Weijun Hong, Zeyi Kang, Xianwei Wang, Feng Wang, En Wang

**Affiliations:** 1 Department of Pharmacy, Taizhou Hospital of Zhejiang Province affiliated to Wenzhou Medical University, Linhai, Zhejiang, China; 2 Taizhou Key Laboratory of Pharmaceuticals Therapy and Translation Research, Linhai, Zhejiang, China; 3 School of Pharmacy, Wenzhou Medical University, Wenzhou, Zhejiang, China; 4 Department of Neurology, Taizhou Hospital of Zhejiang Province affiliated to Wenzhou Medical University, Linhai, Zhejiang, China

**Keywords:** imaging readouts, ischemic stroke, microcirculation dysfunction, pathomechanism, therapeutic strategy

## Abstract

Early, effective reopening of an occluded large artery is a cornerstone of modern ischemic stroke care. Yet angiographic success does not consistently translate into tissue level reperfusion, nor does it reliably forecast good neurological recovery. Futile recanalization is multifactorial, and in many patients poor outcome may also reflect extensive irreversible injury already present at the time of recanalization because reopening is achieved too late to rescue substantial tissue. A growing body of work suggests that persistent microcirculatory dysfunction after recanalization may represent one important contributor to this discordance. In the post-recanalization setting, microvascular failure reflects intertwined ischemia-reperfusion processes: oxidative stress, inflammatory activation, endothelial injury, microthrombosis, and pericyte-driven capillary constriction, that together degrade capillary perfusion and exchange. As a result, tissue hypoxia can persist and the infarct core may continue to expand even when large-vessel patency has been restored. Experimental studies and emerging clinical observations, including trials of adjunct intra-arterial thrombolysis, increasingly support the idea that resolving residual microvascular obstruction can yield functional benefits beyond macrorecanalization alone. In parallel, progress in microcirculation imaging and monitoring, ranging from high-resolution optical tools in experimental models to angiography- and perfusion-derived metrics in patients, has strengthened *in vivo* evaluation of microvascular reperfusion and treatment effects. This review integrates current detection approaches, mechanistic drivers across microvascular segments, and translational therapeutic strategies, and it outlines priorities for clinically practical microcirculatory readouts and imaging-guided precision trials to speed development of microvascular-targeted adjunct therapies for ischemic stroke.

## 1 Introduction

Ischemic stroke is a leading cause of death and disability worldwide, most often caused by acute arterial occlusion that abruptly reduces cerebral blood flow (GBD 2019 Stroke Collaborators, 2021; Zhao et al., 2026)and increasingly affects younger adults (Zhang et al., 2025). Cerebral ischemia rapidly induces metabolic failure, membrane depolarization, ion-channel dysfunction, and ATP depletion, ultimately leading to irreversible neuronal injury if perfusion is not restored (Jordan et al., 2011; Kalogeris et al., 2012). Intravenous thrombolysis and endovascular thrombectomy are established recanalization therapies (Popiela et al., 2022), but their benefits remain limited by narrow time windows, treatment-related complications, and poor outcomes despite successful angiographic recanalization (Goyal et al., 2016; Wei et al., 2020; Mujanovic et al., 2025a; Peña et al., 2017), a phenomenon termed futile recanalization (FR) (Meinel et al., 2022).

Although baseline infarct core, collateral status, and ischemia duration are key determinants (Meinel et al., 2022; Wang and Xiong, 2023; Shuaib et al., 2011; Rafols, 2015; El et al., 2020), growing evidence also implicates microcirculatory dysfunction in cerebrovascular disorders (Staehr et al., 2023; Chow et al., 2020). The cerebral microcirculation, comprising arterioles, capillaries, and postcapillary venules with luminal diameters typically <20 μm, is the terminal vascular network responsible for oxygen and nutrient delivery and is essential for neuronal metabolism and neurovascular coupling (Guven et al., 2020; Yang Q. et al., 2022). Disruption of this network through microthrombotic embolism, capillary narrowing, sluggish flow, leukocyte adhesion, or microthrombosis can impair tissue reperfusion despite macrovascular reopening. Given the limited clinical translation of neuroprotective strategies (Paul and Candelario-Jalil, 2021; Zaleska et al., 2009), targeting microcirculatory failure may provide a promising approach to improve recovery after ischemic stroke and recanalization therapy.

## 2 Microcirculation dysfunction and FR

**FIGURE 1:**
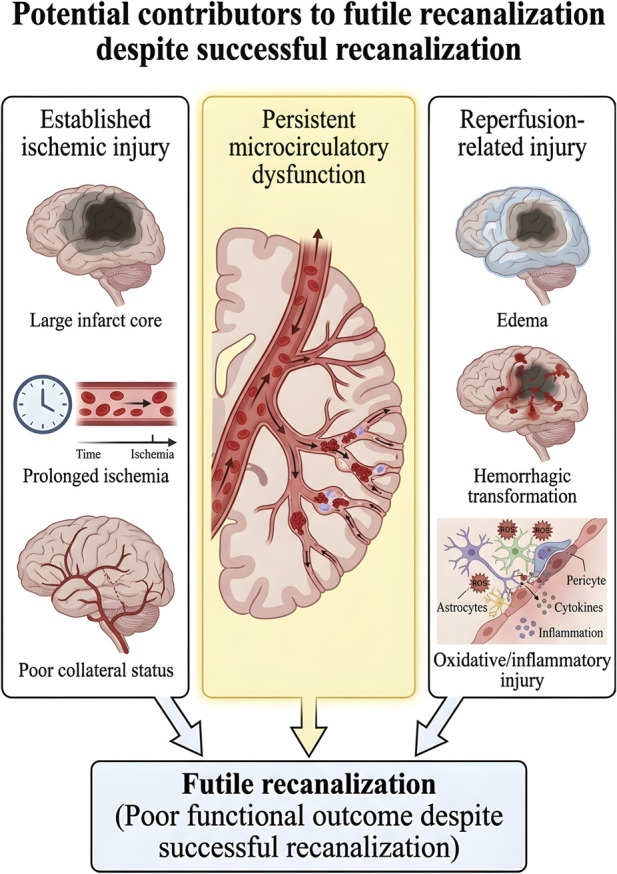
Major contributors to futile recanalization. Poor functional outcome despite successful recanalization may result from several interacting processes, including established ischemic injury, persistent microcirculatory dysfunction, and reperfusion-related injury. Because microcirculatory dysfunction is a major focus of this review, its detailed mechanisms are presented separately in Figure 2.

FR after technically successful endovascular therapy (EVT) is likely multifactorial. In some patients, poor outcome may primarily reflect extensive irreversible injury already present before recanalization, particularly when baseline infarct core is large or treatment is achieved late. In others, persistent microcirculatory dysfunction after recanalization may further limit tissue-level reperfusion and contribute to incomplete neurological recovery. Even when the occluded large artery is reopened, capillary-level flow can remain severely compromised, leaving tissue hypoperfused. Clinically, a 2026 systematic review and meta-analysis including eight studies (n = 1483) reported FR in 20.5% of EVT-treated patients and found strong associations with reduced early neurological recovery (RR 0.76) and higher hemorrhagic complications, including hemorrhagic transformation (RR 1.82) and symptomatic intracerebral hemorrhage (RR 1.88) (Pereira da Silva et al., 2026). Together, these findings support an association between post-recanalization microvascular failure and poorer early neurological recovery as well as hemorrhagic complications (Figure 1).

Microcirculatory impairment is also bidirectionally linked to reperfusion itself. Ischemia–reperfusion can disrupt the blood–brain barrier, promote vasogenic edema, amplify inflammatory–thrombotic signaling, and trigger secondary microvascular constriction, forming a self-reinforcing cycle in which initial capillary compromise fuels further obstruction and tissue injury. Mechanistic *in vivo* studies show that delayed recovery of capillary diameter by surviving cortical pericytes predicts subsequent microvascular reconstriction, offering a plausible cellular basis for this vicious loop (Shrouder et al., 2024).

The prognostic and therapeutic relevance of the microcirculation is further illustrated by adjunct intra-arterial thrombolysis trials intended to extend reperfusion beyond macrovascular reopening. In the CHOICE trial, intra-arterial alteplase administered after successful thrombectomy increased the proportion of patients achieving excellent functional outcomes at 90 days (modified Rankin Scale [mRS] 0–1: 59.0% vs. 40.4%) (Renú et al., 2022). A larger multicenter randomized controlled trial similarly reported improved excellent outcomes (44.8% vs. 30.2%) (Writing Committee for the PEARL Investigators, PEARL, 2025). Taken together, these results support the idea that addressing residual microvascular obstruction can yield meaningful functional gains beyond large-vessel recanalization alone (Barbagallo et al., 2025).

## 3 Detection of cerebral microcirculation and microvascular No-Reflow

FR reflects the dissociation between macrovascular reopening and tissue-level reperfusion, accurate assessment of residual microcirculatory dysfunction is essential in both experimental and clinical studies of post-recanalization stroke. Yet the lack of a robust, standardized clinical phenotype remains a major translational bottleneck, limiting patient stratification, mechanistic interpretation, and targeted therapeutic development. This section summarizes contemporary approaches for evaluating microcirculatory function across preclinical and clinical settings.

Compared with peripheral microvascular beds (e.g., skin, muscle, intestinal mucosa), intracranial microcirculation poses distinctive technical barriers. Skull related attenuation and scattering, limited optical access, and depth dependent signal mixing make direct visualization of cortical and subcortical microvessels challenging. As a result, optical and acoustic techniques have been adapted to probe cortical microvascular structure, flow related signals, and oxygenation, while radiological modalities offer whole brain surrogates of perfusion and metabolic sufficiency. Notably, each method captures different physiological dimensions: blood flow, blood volume, oxygenation, or metabolism, across distinct spatial and temporal scales. These constraints must be considered explicitly when interpreting evidence for “microvascular reperfusion” or microvascular no-reflow after recanalization.

In this review, we use the term microvascular no-reflow to describe persistent failure of effective microvascular reperfusion after successful upstream arterial recanalization. Ideally, this corresponds to absent or severely impaired capillary red-blood-cell passage at the tissue level; however, in clinical practice it is often inferred indirectly from perfusion, contrast transit, or metabolic surrogates.

### 3.1 *In Vivo* assessment

A key difficulty in evaluating post-recanalization microcirculatory dysfunction is that reopening a large vessel does not reliably predict capillary reperfusion. *In vivo* techniques can therefore be meaningfully compared not only by their imaging principles but also by practical, functionally relevant attributes: the smallest vascular segment that can be interrogated (capillaries versus arterioles/venules); achievable imaging depth and field-of-view; temporal resolution and suitability for longitudinal measurement; invasiveness, including skull manipulation, contrast agents, or ionizing radiation; and the capacity to distinguish blood flow metrics (CBF, velocity, or flux) from blood volume (CBV). This latter distinction is particularly important when interpreting microvascular failure and ineffective tissue reperfusion after recanalization (Dunn, 2012; Martinez de Paz and Macé, 2021; Hellmann et al., 2022; Takasaki et al., 2019; Yang et al., 2025).

#### 3.1.1 Optical micro- and mesoscopic techniques

##### 3.1.1.1 Optical access as an enabling strategy

Cranial window and thinned-skull preparations are best viewed as enabling strategies rather than independent modalities. By reducing skull-induced scattering and attenuation, these approaches allow stable, repeated cortical imaging with Optical coherence tomography (OCT), two-photon imaging (TPI), laser speckle contrast imaging (LSCI), photoacoustic imaging (PAI), and optical intrinsic signal imaging (OISI), supporting longitudinal experiments spanning weeks to months (Wang and Xi, 2021; Yeon et al., 2022). This repeatability and compatibility across modalities are clear strengths. However, cranial access is intrinsically invasive and can perturb local physiology through inflammation, edema, or altered intracranial pressure. Such potential confounding effects should be acknowledged when apparent microcirculatory recovery is interpreted.

##### 3.1.1.2 OCT and Doppler-OCT

OCT provides label-free, depth-resolved three-dimensional reconstruction of microvascular architecture at capillary-level resolution and enables quantification of perfused vessel density (Wang and Xi, 2021). Doppler-based variants further estimate axial blood-cell velocity and flow direction within microvessels (Hohberger et al., 2021; Gao, 2018; Shin et al., 2019; Li et al., 2018) and flow direction results can partially support arterial–venous differentiation (e.g., positive versus negative axial velocity under defined geometry) (Srinivasan et al., 2012). Despite these powerful features, practical limitations remain for human *in vivo* applications, particularly in intracranial settings. One key restricting factor has been the stiffness of the probe, making it very difficult to navigate into the intradural vasculature of the brain. Recent advances, such as the development of neuro-OCT technology (Lopes et al., 2026), have partially overcome this issue, improving the flexibility and maneuverability of OCT probes for intracranial use. In addition to probe flexibility, other challenges include restricted imaging depth and field-of-view due to tissue scattering, which often necessitates cranial access. In addition, quantitative flow estimates depend strongly on vessel orientation and signal-to-noise characteristics, which limit robust absolute flow measurement in complex cortical networks (Yang et al., 2025). As a result, OCT and Doppler-OCT are particularly useful for evaluating microvascular patency and structural remodeling rather than cellular-scale mechanisms of capillary stalling (Introduction to Multiphoton Fluorescence Microscopy). Accordingly, both of them can partially define microvascular no-reflow by demonstrating loss of perfused capillary segments or markedly reduced microvascular flow, but their ability to establish true tissue-wide no-reflow is constrained by limited depth, field-of-view, and challenges in absolute flow quantification.

##### 3.1.1.3 Two- and three-photon imaging

Two-photon imaging (TPI) enables direct measurement of red-blood-cell velocity and flux in individual capillaries and allows visualization of leukocyte–endothelial interactions that contribute to capillary stalling (Kim et al., 2019). With submicron spatial resolution and penetration depths approaching ∼600 μm, TPI offers exceptional insight into capillary-level no-reflow dynamics (Takasaki et al., 2019; Shih et al., 2013). These advantages make TPI one of the strongest experimental tools for directly defining microvascular no-reflow at the capillary level, particularly through visualization of red-blood-cell stalling, capillary transit failure, and leukocyte-associated obstruction. However, its interpretation remains limited by small fields-of-view, restricted penetration, substantial technical demands, and the need for invasive optical access. Like TPI, three-photon imaging (3PI) may directly detect capillary-level no-reflow in deeper cortical layers, but its current application remains largely experimental and spatially restricted (Giblin et al., 2023; Horton et al., 2013; Evident, 2024).

##### 3.1.1.4 Laser speckle contrast imaging

LSCI is used mainly in preclinical stroke models and occasionally as an intraoperative adjunct for cortical perfusion monitoring. It provides wide-field, high temporal resolution maps of relative surface perfusion, allowing continuous assessment of reperfusion dynamics and spatial heterogeneity. Its simple, cost-effective setup supports repeated translational measurements. However, LSCI is limited to superficial tissue, lacks true depth resolution (∼300 μm), is affected by depth-mixing, and provides a relative flow index rather than absolute CBF. It also does not reliably distinguish flow from microvascular blood volume. Accordingly, LSCI is useful for identifying surface hypoperfusion suggestive of no-reflow, especially in experimental cortex, but it cannot directly define capillary-level or deep microvascular no-reflow because it lacks depth resolution and provides only a relative flow index (Dunn, 2012; Hellmann et al., 2022).

##### 3.1.1.5 Photoacoustic and ultrasound-based approaches

Photoacoustic imaging integrates optical absorption contrast with ultrasound detection, enabling visualization of microvascular structure and oxygenation-related signals at depths beyond those achievable with purely optical methods (Wang and Xi, 2021; Mirg et al., 1994; Liu and Wang, 2022). As such, photoacoustic imaging may provide indirect evidence of no-reflow through persistent microvascular deoxygenation or absent perfusion-related signals, but it does not directly resolve capillary red-blood-cell transit failure. In practice, skull attenuation and the need for specialized cranial windows limit routine use, particularly in human studies (Yang et al., 2021). Ultrafast ultrasound and ultrasound localization microscopy permit deep transcranial imaging in small animals and generate high-quality perfusion maps (Binder et al., 2024; Hingot et al., 2020). Many functional ultrasound readouts predominantly reflect CBV changes rather than capillary red-blood-cell flow, which is an important interpretive distinction in studies of microvascular no-reflow (Martinez de Paz and Macé, 2021).

##### 3.1.1.6 Multimodal optical systems

Multimodal optical platforms that combine approaches such as TPI, OCT, and LSCI can concurrently probe blood flow, oxygenation, and metabolic signals, supporting integrated assessment of neurovascular coupling and microcirculatory status (Yaseen et al., 2015). Their use remains largely confined to specialized laboratories because of cost and technical complexity. Optical intrinsic signal imaging (OISI), particularly when paired with LSCI, can provide indirect estimates of cerebral metabolic rate of oxygen (CMRO_2_), though spatial specificity remains limited (Sintsov et al., 2017). By integrating complementary readouts of flow, oxygenation, and metabolism, multimodal optical systems may strengthen confidence in the presence of microvascular no-reflow; however, whether no-reflow is defined directly or only inferred still depends on the specific components included in the platform.

##### 3.1.1.7 Sublingual microcirculation

Sublingual microcirculation imaging using side-stream dark-field technology or GlycoCheck offers a practical, non-invasive way to visualize microvessels <30 μm and quantify capillary density and flow patterns (Khalilzada et al., 2011). Although this approach is feasible at the bedside, current evidence is insufficient to support sublingual imaging as a valid method for detecting or defining intracranial microvascular no-reflow, and it should therefore be regarded as a systemic surrogate at best (Wadowski et al., 2022).

#### 3.1.2 Radiological approaches

Radiological modalities, including Computed Tomography (CT) and Magnetic Resonance Imaging (MRI), remain central to clinical evaluation after stroke recanalization (Regenhardt et al., 2023). Digital subtraction angiography (DSA) can suggest downstream no-reflow through delayed capillary phase filling, prolonged microcirculatory transit, or reduced tissue blush despite proximal recanalization, but it remains an indirect angiographic surrogate rather than a direct capillary-level measure (Ji et al., 2023). CT and MR perfusion can support identification of persistent tissue hypoperfusion after recanalization, which is clinically relevant to no-reflow, but they do not directly visualize capillary obstruction or red-blood-cell stalling and are therefore indirect, model-dependent surrogates. Susceptibility-weighted imaging may reveal venous prominence, susceptibility vessel signs, or distal thromboembolic/microembolic phenomena relevant to impaired reperfusion, but its relation to microvascular no-reflow is indirect and potentially confounded by hemorrhage, deoxygenation, or venous congestion (Regenhardt et al., 2023; Wong et al., 2021; Tang et al., 2021). Positron Emission Tomography (PET) adds metabolic specificity by measuring CMRO_2_ and oxygen extraction, but high cost and logistical barriers limit widespread use (Engle et al., 2024). PET can identify metabolic consequences consistent with no-reflow, such as impaired oxygen metabolism or persistent oxygen extraction abnormalities, but it does not directly define capillary non-perfusion and is therefore best viewed as a downstream metabolic correlate. Overall, radiological tools provide indispensable whole-brain coverage, while segment-level microvascular dysfunction is typically captured only indirectly and at limited resolution.

### 3.2 *Ex Vivo* detection methods

*Ex vivo* methods deliver high-specificity structural confirmation of microvascular obstruction but cannot capture dynamic flow behavior. Lectin based labeling supports precise quantification of obstructed vessels and is particularly useful for confirming non-perfused microvascular segments and quantifying the extent of structural no-reflow at endpoint (Li et al., 2023). Tissue clearing enables three-dimensional reconstruction of occlusion patterns and microvascular network disruption, but only as a terminal structural correlate of no-reflow (Ma et al., 2023). Therefore, they can validate the anatomical substrate of no-reflow (e.g., non-patent versus patent microvessels) but cannot independently define functional no-reflow in real time.

### 3.3 Synthesis and perspective

Across modalities, an enduring gap persists between imaging defined reperfusion and true recovery of microcirculatory function, particularly when the target phenotype is microvascular no-reflow rather than macrovascular reopening alone. Only a limited subset of techniques can directly interrogate capillary-level no-reflow, whereas most clinically available modalities infer it indirectly from perfusion, contrast transit, or metabolic consequences. This divergence reflects fundamental differences in physiological contrast, spatial resolution, and temporal sensitivity. Closing the recanalization and reperfusion mismatch will likely require integrative, multimodal strategies that combine complementary measures of flow, volume, and oxygen utilization, rather than relying on any single surrogate of microcirculatory status.

Table 1 summarizes commonly used experimental and clinical techniques for assessing cerebral microcirculation after stroke recanalization, organized by typical use context (and translational feasibility), measurable outputs, advantages, limitation (invasiveness), cost-effectiveness and “flow vs. volume discrimination”. The dimensions of cost-effectiveness and “flow vs. volume discrimination” are conceptual and not based on standardized methodological comparisons. Techniques differ fundamentally in whether they primarily reflect capillary-level blood flow (velocity or flux), CBV, oxygenation, or metabolism, which directly affects interpretation of “microvascular reperfusion.” Microscopy-based approaches (e.g., TPI/3PI, OCT/Doppler-OCT) provide high microvascular specificity but are largely confined to experimental settings, whereas radiological methods such as CT/MR perfusion, DSA, PET offer whole-brain coverage but infer microcirculatory dysfunction indirectly through model-dependent surrogates. Mesoscopic techniques (functional ultrasound, photoacoustic imaging) provide intermediate depth and temporal resolution, yet often yield CBV- or oxygenation-weighted signals rather than direct measures of capillary flow. An additional column indicates whether each technique can assess microvascular no-reflow directly, only indirectly through surrogate measures, or not in a validated manner.

**TABLE 1:** Imaging and assessment techniques for cerebral microcirculation after stroke recanalization.

Modality/ strategy ^Refs^	Typical use context (and translational feasibility)	Key measurable outputs	Advantages	Cost-effectiveness	Key limitations	Flow vs. volume discrimination	Ability to assess microvascular no-reflow
cranial window/ thinned skull (Wang and Xi, 2021; Yeon et al., 2022)	Preclinical longitudinal studies	Enables stable repeated imaging of cortical microvessels	Maximizes repeatability; supports multimodal combinations	Medium	Invasive; potential physiological perturbation; mainly cortex	Depends on paired modality	Not a detection modality itself
OCT/OCTA (Yang et al., 2025; Hohberger et al., 2021; Gao, 2018; Shin et al., 2019; Li et al., 2018)	Preclinical microvascular mapping; Preclinical to early translational	Depth-resolved microvascular structure; perfused vessel density (capillary-level networks)	Label-free, high-resolution 3D microvessel imaging	Medium	Imaging depth/FOV constrained by scattering/skull; often benefits from optical access; probe stiffness	Mostly flow-presence/structure; velocity needs Doppler variants	Direct/partial in accessible cortex
Doppler-OCT (Srinivasan et al., 2012; Introduction to Multiphoton Fluorescence Microscopy; Leitgeb et al., 2014; Yang et al., 2003)	Preclinical flow mapping; Preclinical to early translational	Axial velocity component from phase shifts; flow direction/relative velocity (geometry dependent)	Adds flow direction/velocity sensitivity to OCT	Medium	Doppler-angle uncertainty; SNR-dependent bias; absolute quantification challenging	Flow-weighted (velocity component); arterial and venous discrimination	Direct/partial
TPI (two-photon) (Takasaki et al., 2019; Introduction to Multiphoton Fluorescence Microscopy; Kim et al., 2019; Shih et al., 2013)	Preclinical capillary mechanisms Research-only	Single-vessel RBC velocity/flux; leukocyte–endothelium interactions	Strongest for capillary-level “no-reflow” mechanisms	Low	Depth fundamentally limited by scattering (∼balance depth ∼600 μm typical); small FOV	**Flow-weighted** (RBC velocity/flux); arterial and venous discrimination	Direct capillary-level assessment
3PI (three-photon) (Giblin et al., 2023; Horton et al., 2013)	Preclinical deeper imaging Research-only	Deeper microvascular imaging than TPI	Better depth/SBR than TPI; can extend below superficial cortex	Low	Very high cost/complexity; depth can reach to 1.4 mm	Flow-weighted; arterial and venous discrimination	Direct capillary-level assessment
LSCI (Dunn, 2012; Hellmann et al., 2022)	Preclinical/ intraoperative adjunct	Wide-field relative perfusion index; real-time dynamics	High temporal resolution; simple setup	High	Superficial/no depth resolution; depth ∼300 μm; relative (not absolute) CBF	**Flow-index** (relative)	Indirect
PAI (Wang and Xi, 2021; Mirg et al., 1994; Liu and Wang, 2022; Yang et al., 2021)	Preclinical oxygenation-relevant; Emerging translational	Hemoglobin-contrast; oxygenation surrogates	Adds oxygenation relevance beyond pure flow mapping	Medium–High	Skull barrier; often needs special window; translation difficult	Often volume/oxygenation-weighted	Indirect
Ultrafast/ functional ultrasound (fUS) (Martinez de Paz and Macé, 2021; Binder et al., 2024; Hingot et al., 2020)	Preclinical deep/mesoscopic monitoring; High translational potential	Power Doppler changes,**CBV**; high spatiotemporal resolution (∼100 × 110 × 300μm^3^; up to ∼10 Hz reported)	Deep penetration; large coverage; high temporal sensitivity	Medium	Often CBV-weighted; small-vessel RBC velocity/CBF estimation challenging	Volume-weighted (CBV proxy)	Indirect
OISI (±LSCI) (Sintsov et al., 2017)	Preclinical hemodynamics	Hemodynamic/metabolic signals over large cortex	Wide coverage; useful for coupling analyses	Medium	Low spatial resolution; indirect microvascular inference	Indirect	Indirect
Multimodal optical system (Yaseen et al., 2015)	Advanced preclinical; Research-only	Flow + pO_2_ + NADH, etc.	Rich multi-parameter interpretation	Low	Complex integration/operation	Both (multi-output)	Depends on components
SDF/GlycoCheck Khalilzada et al. (2011) Wadowski et al. (2022)	Bedside surrogate screening; Clinically feasible but unvalidated	Microvessels <30 μm; functional capillary density; flow patterns	Non-invasive; easy operation	High	Intracranial validity uncertain	Flow pattern (surrogate)	Not validated for intracranial no-reflow
DSA (Ji et al., 2023; Singer et al., 2025)	Established clinical tool	mCCT, contrast transit delay, capillary phase filling, tissue blush intensity	Excellent temporal resolution; evaluation of downstream microcirculatory reperfusion	Medium–Low	Invasive; indirect assessment of capillary perfusion	Flow-dominant; arterial and venous discrimination	Indirect surrogate
CTP/ perfusion MRI (Regenhardt et al., 2023; Mujanovic et al., 2023; Mujanovic et al., 2025b; N et al., 2022; Dai et al., 2013; Lubo et al., 2020)	Established clinical standard	Regional perfusion (MTT), Tmax, residual hypoperfusion volume	Whole-brain coverage; workflow-ready	Medium–Low	Radiation/contrast (CTP/PET); model-dependent quantification	Often **CBF + CBV** (model-based)	Indirect surrogate
SWI (Wong et al., 2021; Tang et al., 2021)	Clinical embolization, microvascular obstruction detection	Presence, number, and spatial distribution of SVS	Non-invasive; whole-brain coverage; sensitive to thrombus -related magnetic susceptibility	High	Indirect marker; venous congestion and hemorrhage confounding	Neither directly	Indirect/supportive only
PET-CT (Engle et al., 2024)	Preclinical or Clinical	metabolic surrogates (CMRO2 and OEF)	Whole-brain coverage; workflow-ready	Low	high cost	Metabolism	Indirect
Lectin labeling (*ex vivo*) (Li et al., 2023)	Preclinical endpoint; Non-translational	Obstructed vs. patent microvessels (3D identification)	High specificity for obstruction quantification	Medium	Endpoint only; no dynamics	N/A	Structural endpoint only
Tissue clearing + optical imaging (*ex vivo*) (Ma et al., 2023)	Preclinical endpoint 3D mapping; Non-translational	3D vascular network and occlusion trajectories	Complete network visualization	Medium	Complex processing; no dynamics; possible distortion	N/A	Structural endpoint only

Abbreviations: OCT, optical coherence tomography; OCTA, optical coherence tomography angiography; Doppler-OCT, doppler optical coherence tomography; TPI, two-photon imaging; 3PI, three-photon imaging; LSCI, laser speckle contrast imaging; PAI, photoacoustic imaging; fUS, functional ultrasound; ULM, ultrasound localization microscopy; OISI, optical intrinsic signal imaging; CT, computed tomography; DSA, digital subtraction angiography; mCCT, microvascular cerebral circulation time; CTP, computed tomography perfusion; MTT, mean transit time; MRI, magnetic resonance imaging; SWI, Susceptibility-Weighted Imaging; SVS, susceptibility vessel signs; PET, positron emission tomography; CMRO_2_, cerebral metabolic rate of oxygen; OEF, oxygen extraction fraction; CBF, cerebral blood flow; CBV, cerebral blood volume; CMRO_2_, cerebral metabolic rate of oxygen; OEF, oxygen extraction fraction; RBC, red blood cell; SDF, sidestream dark-field; NADH, nicotinamide adenine dinucleotide (reduced form); FOV, field of view; SNR, signal-to-noise ratio.

Collectively, while imaging advances have expanded our ability to probe downstream perfusion, the lack of standardized, validated, and clinically feasible measures of microcirculatory dysfunction remains a major barrier to both mechanistic understanding and therapeutic translation. Importantly, these microcirculatory readouts should be interpreted within a well-defined baseline tissue-status context, particularly regarding the presence of salvageable tissue.

## 4 Pathogenesis of microcirculatory dysfunction in ischemic stroke

Recanalization after prolonged ischemia is not uniformly beneficial, and ischemia–reperfusion (I/R) injury is common across multiple ischemic diseases (Francis and Baynosa, 2017). In ischemic stroke, accumulating evidence indicates that microcirculatory dysfunction after recanalization represents a central pathological mechanism limiting effective tissue reperfusion and functional recovery (Jiang et al., 2022; Hu et al., 2023). Multiple intertwined processes contribute to post-recanalization microcirculation failure including distal microembolic/microthrombotic obstruction (Chen et al., 2021), ischemia- and reperfusion-driven oxidative stress with mitochondrial dysfunction and excessive free-radical generation (Shademan et al., 2023), and downstream inflammatory activation, endothelial dysfunction, and pericyte dysregulation (Jiang et al., 2022). These mechanisms can begin early during ischemia, intensify with longer ischemic duration, and be further amplified by reperfusion, ultimately sustaining microvascular obstruction. Preventing or reversing this microcirculatory failure, even when macrovascular recanalization appears satisfactory, remains a major barrier to improving stroke outcomes.

### 4.1 Microembolic/microthrombotic embolism

Distal embolization represents a distinct and clinically relevant mechanism of downstream hypoperfusion after EVT. Fragmentation and migration of thrombus during or prior to thrombectomy can occlude distal arterial and arteriolar branches, leading to perfusion deficits that may mimic or coexist with intrinsic microcirculatory dysfunction. Importantly, this mechanism is mechanistically and therapeutically distinct from capillary-level no-reflow. The former reflects impaired inflow due to upstream vascular occlusion, whereas the latter represents dysfunction at the capillary level despite adequate arterial patency. After thrombectomy, susceptibility-weighted imaging (SWI) identifies embolic phenomena in approximately 42% of patients, including emboli into new territories (ENT) in ∼22% and emboli into distal territory (EDT) in ∼20%. These distributions are consistent with distal trapping of embolic material within arterial and microvascular beds and are expected to contribute to incomplete tissue reperfusion (Wong et al., 2021; Chen et al., 2021). Angiographic indices reinforce this mechanism: patients with impaired microcirculatory reperfusion show prolonged microvascular circulation time (mCCT) on digital subtraction angiography (DSA) (e.g., 3.22 ± 0.85 s versus 2.79 ± 0.64 s), consistent with delayed microvascular transit due to distal obstruction (Ji et al., 2023; Singer et al., 2025). At the tissue level, microembolization-related microcirculatory failure can also manifest as residual perfusion deficit volume on post-procedural CT/MR perfusion despite successful large-vessel recanalization (expanded Thrombolysis in Cerebral Infarction (eTICI) 2c/3)^66^.

### 4.2 Reactive species

Oxidative stress follows a distinct temporal profile during ischemia–reperfusion. Mitochondrial dysfunction contributes to metabolic disruption during ischemia, whereas the reintroduction of oxygen at reperfusion can trigger a sudden burst of reactive species (reactive oxygen species, ROS/reactive nitrogen species, RNS), leading to reperfusion injury characterized by severe endothelial dysfunction and irreversible enzymatic inactivation (Jordan et al., 2011; Kalogeris et al., 2012; Yu et al., 2019; Tao et al., 2014). After ischemia/reperfusion, excessive calcium influx may overwhelm mitochondrial buffering capacity and further compromise mitochondrial function (Jordan et al., 2011)˒^5^. Restoration of molecular oxygen availability markedly increases reactive species derived from oxygen and/or nitric oxide (Yu et al., 2019). Cerebral microvascular endothelial cells are pivotal regulators of cerebral blood flow, shaping vascular tone in resistance vessels, preserving barrier integrity in capillaries and postcapillary venules, and orchestrating proinflammatory/prothrombotic signaling (Yang Q. et al., 2022; Yu et al., 2019). Excess nitric oxide and superoxide drive peroxynitrite formation, which irreversibly inactivates prostacyclin synthase and oxidizes tetrahydrobiopterin, uncoupling endothelial nitric oxide synthase and shifting it toward superoxide production—thereby compounding endothelial dysfunction (Tao et al., 2014). Functionally, reactive species–associated microcirculatory injury most consistently presents as impaired capillary perfusion, increased capillary stall rates, heterogeneous RBC velocity/flux, and reduced microvascular patency, as measured by *in vivo* two-photon microscopy or speckle/OCT-based flow-sensitive imaging, often accompanied by markers of oxidative and nitrative endothelial damage (Taskiran-Sag et al., 2018).

Beyond endothelial impairment, excessive ROS can foster thrombo-inflammatory microvascular occlusion by activating coagulation and complement pathways. Thrombin generation and fibrin deposition then trap erythrocytes, platelets, and polymorphonuclear leukocytes, sustaining microvascular blockage. Experimental work indicates that antioxidant strategies, including vitamin C, can reduce ROS and inhibit platelet aggregation in a concentration-dependent manner (Liu et al., 2020). In parallel, ischemia-reperfusion induced reactive species activate NLRP3 inflammasomes, disturb connexin/pannexin signaling, promote mitochondrial fission, and stimulate endothelial microvesicle release, collectively impairing arteriolar, capillary, and venular function (Yu et al., 2019; Taskiran-Sag et al., 2018).

Reactive species are also context dependent. At lower, signaling-range levels, transient or controlled ischemia–reperfusion and certain pharmacologic stimuli (e.g., ethanol, big-conductance calcium-activated potassium (BKCa) or ATP-sensitive potassium channel (KATP) channel agonists) can induce moderate reactive species production that engages cytoprotective and pro-survival pathways. These adaptive programs may limit excessive reactive species accumulation during later, more prolonged reperfusion and thereby reduce microvascular damage (Kim et al., 2017; Cohen et al., 2016). Collectively, these observations suggest that both the timing and magnitude of reactive species generation shape microcirculatory outcomes after reperfusion.

### 4.3 Inflammation

Inflammatory activation in the cerebral microcirculation begins during ischemia through endothelial activation and upregulation of adhesion molecules, and it intensifies during reperfusion with pronounced leukocyte recruitment and immunothrombosis mediated by neutrophil extracellular traps (NETs) (Fruekilde et al., 2022; Candelario-Jalil et al., 2022; Jayaraj et al., 2019; Ansari and Gavins, 1994; Stoll and Nieswandt, 2019). This reperfusion-phase escalation makes inflammation a major contributor to secondary microcirculatory failure, even when upstream recanalization is achieved.

During inflammation, circulating leukocytes, especially neutrophils, and platelets are recruited in large numbers. Endothelial adhesion molecules, including L-selectin, P-selectin, and intracellular adhesion molecule-1 (ICAM-1), are upregulated on microvascular brain endothelial cells (Bombeli et al., 1998). Activated neutrophils release ROS and NETs rich in procoagulant constituents. NETs bind platelets and promote platelet activation and aggregation, while activated platelets in turn enhance NET release, creating a self-amplifying thrombo-inflammatory circuit that culminates in microthrombus deposition within the reperfused cerebral microvasculature. Beyond their role in downstream microvascular thrombosis, NETs have also been detected in retrieved ischemic stroke thrombi, where they contribute to thrombus architecture through interactions with fibrin and platelet-rich components (Laridan et al., 2017). These observations suggest that NETs may increase thrombus compactness and treatment resistance, including reduced responsiveness to thrombolysis and potentially greater procedural difficulty during thrombectomy (Vandelanotte et al., 2024). In parallel, resident immune cells (microglia and astrocytes) become activated and produce cytokines such as interleukin-1 beta (IL-1β), interleukin-6(IL-6) and tumor necrosis factor-alpha (TNF-α), which disrupt endothelial tight junctions, increase vascular permeability, and aggravate microcirculatory dysfunction and neuroinflammation (Fruekilde et al., 2022; Candelario-Jalil et al., 2022; Jayaraj et al., 2019; Ansari and Gavins, 1994).

A prominent functional consequence of inflammation is impaired capillary flow. Under physiological conditions, capillary luminal diameter is roughly half the diameter of neutrophils, so successful transit requires coordinated deformation of both the cell and the vessel wall. With inflammation, vascular stiffening and glycocalyx degradation undermine this coordination and expose adhesion molecules that encourage leukocyte binding and slow intraluminal passage (Rao et al., 2007; Erdener et al., 2021). The result can be transient or sustained capillary plugging and intravascular congestion, raising capillary stall rates and reducing the fraction of perfused capillaries within the reperfused penumbra. *In vivo* imaging suggests neutrophils can obstruct ∼20%–30% of capillaries after transient ischemia, and neutrophil depletion restores capillary flow by reducing stalls and improving microvascular perfusion, supporting a causal role for leukocyte-mediated obstruction in microcirculatory no-reflow (El et al., 2020).

### 4.4 Pericyte contraction

Pericytes are core elements of the neurovascular unit, residing abluminally along capillaries between endothelial cells and the astrocytic basal lamina. They provide mechanical support to endothelial cells and contribute to blood–brain barrier integrity. Whereas smooth muscle cells govern tone in larger resistance vessels, pericytes act as distal effectors in microvessels that lack smooth muscle coverage by modulating capillary diameter through contraction and relaxation (Alarcon-Martinez et al., 2021; Oztop-Cakmak et al., 2017). Pericytes can also adopt context-dependent inflammatory roles and express high levels of platelet-derived growth factor receptor-β (PDGFR-β), enabling responsiveness to endothelial PDGF-B and participation in angiogenesis and vascular remodeling (Oztop-Cakmak et al., 2017; Cai et al., 2017).

In early ischemia, transient pericyte relaxation has been proposed as a compensatory mechanism to help preserve microvascular perfusion. Endothelial PDGF-B release may facilitate dilation, while adenosine-dependent A2A receptor activation with subsequent KATP channel opening can promote capillary expansion; enhanced nitric oxide signaling via the NO–guanylate cyclase pathway may further contribute (Cai et al., 2017). With sustained ischemia, however, these compensations often fail and pericytes may transition toward persistent contraction. This contraction produces focal capillary narrowing and is increasingly recognized as a major driver of microcirculatory no-reflow during ischemia and after reperfusion (Staehr et al., 2023; Zhang et al., 2022).

Oxidative stress is a strong trigger of pathological pericyte contraction, disrupting vasodilatory signaling and acting as a dominant injurious mediator (Dalkara and Alarcon-Martinez, 2015; Gürsoy-Ozdemir et al., 2004; Kaul et al., 2023). Although direct *in vivo* quantification of pericyte contractile dynamics remains difficult, converging evidence shows increased numbers of pericytes associated with constricted capillaries about 6 hours after cerebral ischemia/reperfusion, accompanied by mechanical restriction of erythrocyte passage and RBC entrapment in ischemic regions. Suppressing oxidative–nitrative stress can restore pericyte function and capillary blood flow, supporting a causal connection between pericyte contraction and impaired microvascular reperfusion (Yemisci et al., 2009; Kloner et al., 2018). Pericyte-mediated no-reflow is characterized by persistent capillary narrowing near pericyte somata, RBC flow arrest or entrapment, and shortened perfused capillary length after recanalization; these features have been quantified with high-resolution *in vivo* imaging, and longitudinal work indicates that a substantial subset of pericytes remain constricted deep into reperfusion, maintaining microvascular hypoperfusion (Shrouder et al., 2024).

In addition, impaired myogenic vasomotion of upstream arterioles can contribute to microcirculatory failure after ischemia–reperfusion. This vasomotion depends on calcium oscillations driven by coupling between mitochondria and endoplasmic reticulum, which weakens after ischemia–reperfusion. Experimental restoration of mitochondrial–ER coupling with ME-Linker reduces capillary stall rates, increases capillary blood flow, and decreases infarct volume and brain atrophy, suggesting that targeting both arteriolar vasomotion and pericyte-driven capillary constriction may improve effective microcirculatory reperfusion after recanalization (Li et al., 2024).

### 4.5 Lactate and lactylation modifications

During ischemia or ischemia–reperfusion, insufficient microvascular perfusion sustains local hypoxia and shifts cellular metabolism from oxidative phosphorylation toward anaerobic glycolysis, increasing lactate production while impairing clearance and generating a high-lactate microenvironment (Liao et al., 2025). Emerging evidence indicates that lactate is not only a metabolic byproduct but also a signaling molecule that can drive lysine lactylation (Kla) (Gu et al., 2026), a post-translational modification with potential regulatory roles across the neurovascular unit that may contribute to microcirculatory dysfunction.

Mechanistically, lactate-driven lactylation appears to disturb microvascular homeostasis across multiple neurovascular cell types. In astrocytes, lactylation interferes with mitochondrial trafficking and metabolic support, weakening the energy delivery needed for post-ischemic recovery (Zhou et al., 2024). In cerebral microvascular endothelial cells, lactylation perturbs calcium signaling, redox balance, and inflammatory pathways, impairing vasomotor control, increasing BBB permeability, and promoting microthrombus formation (Yao et al., 2023). Through these pathways, hyperlactatemia may aggravate microcirculatory dysfunction and reduce the adequacy of tissue-level reperfusion, producing functional ischemia even after upstream recanalization.

Recent ischemia/reperfusion studies connect lactate/lactylation signaling with greater injury severity and slower recovery, often showing concordance between elevated lactylation in specific neurovascular populations and impaired perfusion readouts such as reduced CBF or persistent perfusion deficits on imaging (Zhou et al., 2024). Lactylation mediated modulation of microglia and other immune cells may further intensify inflammatory activation, worsening the microcirculatory environment and reinforcing a cycle of hyperlactatemia, lactylation, and microvascular failure. These findings position protein lactylation as a plausible molecular integrator linking metabolic dysregulation with sustained microvascular dysfunction after reperfusion and suggest its promise as a precision therapeutic target for improving effective tissue reperfusion in ischemic stroke.

### 4.6 Whole blood viscosity (WBV)

Whole blood viscosity (WBV) captures the internal resistance of blood to flow and is a recognized risk factor and prognostic marker for cardiovascular and cerebrovascular events (Song et al., 2017). Under low flow conditions, such as within narrowed microvessels or distal to an arterial occlusion, WBV becomes a decisive determinant of tissue perfusion. When WBV rises, driven by increased plasma viscosity and reduced erythrocyte deformability, peripheral vascular resistance increases and endothelial dysfunction is promoted, further limiting microvascular flow (Gyawali et al., 2023; Gyawali et al., 2022).

WBV related hemodynamic compromise spans the ischemia/reperfusion continuum. During ischemia, reduced shear stress favors erythrocyte aggregation and raises viscosity; after reperfusion, plasma extravasation, endothelial injury, and increased cellular rigidity can maintain high-viscosity flow, with microvascular sludging that restricts effective tissue perfusion (Sun et al., 2024). Rather than acting in isolation, elevated WBV may magnify pre-existing microvascular obstruction and flow heterogeneity, contributing to incomplete reperfusion even when macrovascular recanalization is achieved. Clinically, WBV associated microcirculatory impairment is often inferred when perfusion parameters (CBF/CBV and transit-time indices) remain discordant with angiographic recanalization (Gyawali et al., 2022). Clinical studies suggest that hemorheology-targeted strategies can improve cerebral perfusion, and agents such as vinpocetine have shown efficacy in ischemic cerebrovascular disease, supporting the potential value of viscosity-modulating adjuncts to reperfusion therapy (Feher et al., 2009).

### 4.7 Cerebral edema

Edema-related compression of the cerebral microcirculation often follows a biphasic course: cytotoxic edema develops during ischemia, and vasogenic edema emerges after reperfusion as the blood–brain barrier becomes disrupted. Together, these processes externally constrain microvascular lumen availability and compromise tissue perfusion after recanalization. However, the relationship between reperfusion and edema is complex. Although reperfusion may exacerbate vasogenic edema in severely injured tissue with marked BBB breakdown, timely and effective reperfusion may also limit edema progression by restoring perfusion, reducing ongoing ischemic injury, and mitigating secondary microvascular damage (Garcia et al., 1994; Ayata and Ropper, 2002; Simard et al., 2007).

Experimental studies indicate that ischemia rapidly induces endothelial and astrocytic swelling. Within 60 min of middle cerebral artery occlusion, astrocytic nuclear diameter increases by ∼23.4%, while microvascular luminal surface area decreases by 35% compared with controls. These structural changes narrow microvascular lumen early and may help explain flow obstruction observed soon after reperfusion (Garcia et al., 1994; Ayata and Ropper, 2002).

Edema arises from combined cytotoxic swelling and transcapillary flux of Na^+^, water, and macromolecules. During ischemia, hypoxia-driven ATP depletion disrupts ATP-dependent transporters such as Na^+^/K^+^-ATPase, promoting intracellular osmotic loading and cellular swelling (Simard et al., 2007). Under physiological conditions, water transport is regulated by aquaporin-4 (AQP4) at astrocytic end-feet when the BBB is intact. After ischemia, microglial activation releases inflammatory mediators that stimulate adjacent astrocytes (Fruekilde et al., 2022; Candelario-Jalil et al., 2022). Microglia-derived cytokines (IL-1β, IL-6, TNF-α) upregulate AQP4 and promote astrocytic end-feet swelling while exacerbating neurovascular inflammation and disrupting endothelial tight junctions, thereby increasing BBB permeability, amplifying vasogenic edema, and further impairing microvascular perfusion after recanalization.

Edema therefore imposes extravascular compression on the microcirculation, shrinking functional lumen diameter and sustaining no-reflow alongside BBB failure. Nevertheless, edema should not be viewed solely as an inevitable consequence of reperfusion. In some patients, including those with large established infarcts, successful reperfusion may still confer benefit and may even attenuate vasogenic edema progression, which has been proposed as one mechanism underlying the benefit of reperfusion despite extensive baseline injury (Broocks et al., 2019). The contribution of edema to post-I/R microcirculatory dysfunction can be evaluated with integrated strategies that pair high-resolution *in vivo* imaging of capillary caliber and perfusion with clinical CT/MR perfusion metrics, demonstrating temporal concordance between edema progression and persistent microvascular failure despite successful macrovascular recanalization (Zhou et al., 2026).

### 4.8 Microvessel segment‐specific pathomechanisms

Post-ischemic microcirculatory dysfunction is increasingly understood as multifactorial and segment specific, with different microvascular compartments contributing in complementary, temporally overlapping ways to tissue-level no-reflow and secondary brain injury even after macrovascular recanalization appears successful (Jia et al., 2024).

Small arterioles govern inflow resistance, perfusion pressure, and rapid redistribution of blood flow through autoregulation. After ischemia/reperfusion, endothelial dysfunction and impaired vasoreactivity at the arteriolar level can misdirect inflow and create heterogeneous downstream perfusion, limiting effective tissue reperfusion (Daher and Payne, 2023; Sheriff et al., 2023). These disturbances are compounded by hemorheological changes: greater viscosity, increased erythrocyte aggregation, and reduced deformability, that elevate arteriolar resistance and further constrain inflow (Gyawali et al., 2022; Velcheva et al., 2025). Excess reactive oxygen species in arteriolar endothelium and smooth muscle decreases nitric oxide bioavailability and blunts vasodilatory capacity, thereby increasing inflow resistance and amplifying downstream perfusion heterogeneity (Iadecola and Davisson, 2008; Faraci, 2006).

Capillaries are the main locus of microcirculatory no-reflow, stalling, and exchange failure. Persistent pericyte contraction after ischemia leads to focal narrowing, RBC trapping, and incomplete reperfusion even after proximal flow is restored (Shrouder et al., 2024). Capillaries also represent a dominant site for leukocyte stalling, where low shear stress, endothelial activation, and leukocyte stiffening favor plugging and intensify flow heterogeneity (El et al., 2020; Rolfes et al., 2021). Capillary obstruction is increasingly regarded as the final common pathway that sustains tissue hypoxia and drives infarct expansion despite angiographic recanalization. Capillary dysfunction also intersects with metabolic dysregulation: lactate accumulation and lactate-driven lactylation signal impaired oxygen delivery and anaerobic shift and may in turn worsen endothelial injury and pericyte contractility during reperfusion (Wei et al., 2025).

Postcapillary venules act as key effectors of thrombo-inflammation, immune recruitment, and BBB disruption (Stoll and Nieswandt, 2019). Leukocyte rolling, adhesion, and transendothelial migration occur predominantly in venules, where low shear stress and strong adhesion molecule expression facilitate inflammatory entry into brain tissue (Lauridsen et al., 2014). Platelet–leukocyte aggregates and fibrin-rich microthrombi can accumulate in postcapillary venules, impairing outflow and propagating distal perfusion failure (Shaik et al., 2021). Venular endothelial injury and junctional disassembly are also major contributors to BBB breakdown, promoting vasogenic edema and increasing risk of hemorrhagic transformation after reperfusion (Mathias et al., 2024).

**FIGURE 2:**
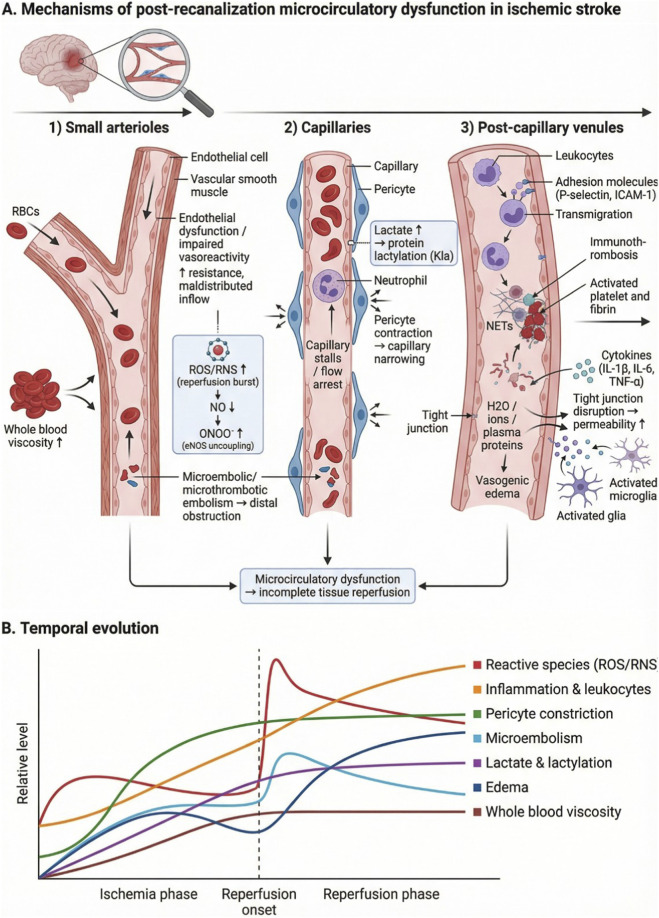
**(A)** Schematic of post-recanalization microcirculatory dysfunction in small arterioles, capillaries, and post-capillary venules. **(B)** Temporal evolution of key determinants of microcirculatory failure from ischemia to reperfusion.

Overall, post-stroke microcirculatory failure reflects coordinated dysfunction spanning arteriolar inflow control, capillary perfusion and exchange, and venular immune–barrier regulation. Within this compartment-specific view, capillary obstruction may represent a key downstream bottleneck linking upstream vascular and inflammatory disturbance to persistent tissue-level hypoperfusion in at least a subset of patients. This framework may help explain why macrovascular recanalization alone does not always translate into neurological recovery and argues for adjunct therapies and imaging endpoints that explicitly target segment-specific drivers of microvascular failure. The segment-specific pathomechanisms and their temporal evolution are summarized in Figure 2.

## 5 Therapeutic strategies and ongoing trials

Cerebral microcirculatory dysfunction following ischemia/reperfusion has moved to the center of discussion as a major determinant of infarct expansion, secondary injury, and neurological outcome, even in an era when macrovascular recanalization can be achieved with high technical success. A wide therapeutic spectrum including antithrombotic, anti-inflammatory, antioxidant, and pericyte-oriented strategies has produced convincing benefits in experimental settings, yet clinical translation remains variable and often disappointing. This section surveys the current preclinical and clinical landscape and highlights a recurring weakness: many mechanistically sophisticated interventions progress without standardized, patient feasible readouts that directly capture capillary level reperfusion failure.

### 5.1 Preclinical strategies

#### 5.1.1 Antiplatelet/ anticoagulant: Microthrombosis is real, but acute microcirculation endpoints are rare

A substantial experimental literature supports platelet activation and microthrombus formation as important contributors to post-recanalization microvascular obstruction. Through platelet–endothelial crosstalk, platelets can intensify local inflammation and facilitate capillary plugging, ultimately compromising downstream perfusion (Gawaz et al., 2005; Kamarova et al., 2022; Gawaz et al., 2023). *In vivo* work also offers a clear causal narrative for platelet-driven no-reflow: in murine focal ischemia–reperfusion, GP IIb/IIIa–dependent platelet aggregation was associated with microcirculatory obstruction, and pharmacologic GP IIb/IIIa inhibition reduced microvascular thrombus burden while improving a microcirculation-relevant perfusion readout (laser Doppler cortical flow ratio at 24 h) (Choudhri et al., 1998). This observation is consistent with a proposed GPIIb/IIIa-dependent bridging mechanism involving fibrinogen/fibronectin/vWF124 and, in turn, supports the logic of short-acting GPIIb/IIIa blockade (e.g., tirofiban) as a microcirculation-oriented adjunct (Zhao et al., 2024) Table 2.

**TABLE 2:** Preclinical research and treatments for microcirculation dysfunction after stroke.

Therapeutic strategy	Drug/ Agent	Mechanism/ Target	Key effects on microcirculation/ Outcomes
Anti-platelet or Anti-coagulant	Aspirin, Clopidogrel, Dipyridamole	COX inhibition, P2Y12 inhibition, etc. (Standard antiplatelet activity)	Widely used for stroke prevention; however, associated with bleeding risks and resistance. Note: Limited specific data on post-stroke microcirculation improvement (Erdener et al., 2021)
	Tirofiban	GPIIb/IIIa receptor inhibitor	Acts as a neuroprotectant potentially by improving microcirculation; inhibits platelet adhesion to ECs (Cai et al., 2017)
	3K3A-APC (Activated Protein C variant)	Cytoprotection via PAR1, PAR3, and EPCR. (Engineered to lack >90% anticoagulant activity) (Dalkara and Alarcon-Martinez, 2015; Gürsoy-Ozdemir et al., 2004)	Exerts direct cytoprotective effects rather than anticoagulant effects; highly tolerated in clinical trials; protects against MCAO injury (Kaul et al., 2023)
Anti-inflammation	Luteolin	TLR4/Myd88/NF-κB signaling pathway	Inhibits expression of adhesion molecules (ICAM-1, VCAM-1); reduces leukocyte adhesion to ECs (Kloner et al., 2018)
	Emodin	PPARγ regulation	Mitigates astrocyte swelling and neuroinflammation; reduces secretion of cytokines (IL-1β, TNF-α, IL-6) (Chen et al., 2024)
	Xueshuantong (XST)	JAK2/STAT3 and NF-κB signaling via JNK.	Inhibits inflammation; increases tight junction proteins (ZO-1, occludin) to repair EC structural damage (Gyawali et al., 2022)
Anti-ROS	Total Salvianolic Acid Injection (TSI)	NADPH oxidase inhibition via AMPK/Akt/PKC pathway	Reduces ROS production by inactivating NADPH oxidase
	NBP-CeO2NPs (Dl-3-n-butylphthalide loaded Cerium oxide nanoparticles)	Free radical scavenging	Combines antioxidant activity with neurovascular repair capabilities (Zhou et al., 2024)
	Melatonin	Nrf2/Keap1 antioxidative signaling pathway	Inhibits ischemia-induced protein nitration of Keap1; protects ECs under nitrosative stress (Tao et al., 2013)
Anti-pericyte Contraction	Iptakalim	K-ATP channel opener (suppresses SUR2/EPAC1 complex)	Reduces Ca^2+^ influx and ET-1 release; inhibits pericyte contraction; decreases the number of obstructed capillaries (Song et al., 2017)
	Fasudil	Rho-kinase inhibitor	Blocks capillary constriction and associated hemodynamic changes (Gyawali et al., 2023)
	TMEM16A Inhibitors	Calcium-activated chloride channel inhibition	Slows pericyte Ca^2+^ rise; reduces capillary constriction, pericyte death, and neutrophil stalling; improves reperfusion (Korte et al., 2022)

Activated protein C (APC) has likewise demonstrated benefit in rodent stroke models (Sheriff et al., 2023). Of particular interest, the engineered 3K3A-APC variant preserves signaling properties while decreasing anticoagulant activity by >90%, implying that the observed protection is driven largely by cytoprotective signaling via PAR1/3 and EPCR (Mosnier et al., 2014; Lyden, 2021; Lyden et al., 2019) rather than by anticoagulation *per se*. Conceptually, this points to an important translational nuance: even within the broad “antithrombotic” category, microvascular protection may be achieved through target engagement that is separable from systemic anticoagulant effects.

#### 5.1.2 Anti-inflammation/anti-ROS: Plausible biology, but often “mechanism ahead of measurement”

Because inflammation promotes both microthrombus formation and leukocyte adhesion after reperfusion (Cao et al., 2023), it creates multiple, biologically credible intervention points. Several compounds have been reported to improve microvascular flow behavior in experimental models: luteolin decreases leukocyte adhesion by downregulating adhesion molecules (ICAM-1/ vascular cell adhesion molecule-1, VCAM-1) through TLR4/MyD88/NF-κB signaling; emodin reduces astrocyte swelling and neuroinflammation via PPARγ-associated cytokine regulation (Su et al., 2021; Chen et al., 2024); and Xueshuantong (Panax notoginseng saponins) influences JAK2/STAT3 and NF-κB through JNK and may help preserve endothelial barrier proteins (ZO-1/occludin) (Wang et al., 2022).

A similar pattern appears in antioxidant approaches, where *in vivo* benefits for microcirculation are frequently reported. Total Salvianolic Acid Injection inhibits NADPH oxidase via AMPK/Akt/PKC and has been linked to improved microvascular perfusion and capillary flow with reduced leukocyte adhesion, based on *in vivo* cerebral microcirculation detection (Tang et al., 1994). Nanoparticle delivery of NBP combined with cerium oxide capitalizes on scavenging and repair-related properties (Li et al., 2022), while melatonin protects endothelial redox signaling through Nrf2/Keap1 by limiting Keap1 nitration under nitrosative stress (Tao et al., 2013). Even so, many of these studies lean heavily on surrogate endpoints with no straightforward clinical counterpart. In practice, translation is constrained less by the biological premise than by the absence of standardized, quantifiable microcirculatory readouts that are feasible in patients, leaving programs in which mechanisms are well articulated but measurement strategies lag behind.

#### 5.1.3 Anti–pericyte contraction: Mechanistically sharp, clinically early

Pericyte contraction offers a direct, capillary level route to microcirculation dysfunction. Iptakalim (a KATP opener) reportedly improves microcirculation by suppressing pericyte contraction through SUR2/EPAC1-linked signaling, lowering Ca^2+^ influx and endothelin-1 release, and reducing obstructed capillaries, with improved perfusion across LSI/multimodal optical/trapped RBC readouts (Guo R. B. et al., 2022). Fasudil (a Rho-kinase inhibitor) prevents optogenetically induced capillary constriction and associated hemodynamic disturbances (Hartmann et al., 2021), and TMEM16A inhibition diminishes pericyte Ca^2+^ elevation, capillary constriction/death, neutrophil stalling, and improves reperfusion in rodent stroke (Korte et al., 2022). Despite their mechanistic precision, these approaches remain early in translational maturity: clinical-grade candidates, optimal therapeutic windows, and human safety profiles are not yet fully established, and trials have rarely incorporated direct capillary-level reperfusion measurements. In short, pericyte-targeted therapy is biologically compelling, but clinically it is still in its formative phase.

### 5.2 Clinical evidence and ongoing trials

#### 5.2.1 Routine practice

Routine post-stroke management includes time-window restricted thrombolysis, antiplatelets (aspirin; clopidogrel/ticagrelor; cilostazol; tirofiban), antioxidant/anti-inflammatory agents (edaravone; edaravone-dexborneol), vasodilators (alprostadil; urinary kallidinogenase), hemorheology modifiers (pentoxifylline; vinpocetine), edema control, and metabolic optimization (D’Anna et al., 2024). Many of these interventions could plausibly influence capillary-level perfusion, yet practical bedside microcirculation detection is still uncommon; as a result, direct clinical evidence for “microvascular reversal” remains limited Table 3.

**TABLE 3:** Clinical research which may be consistent with microcirculatory effects after stroke or recanalization, but no direct evidence is available.

Research ^Ref^	Condition	Treatment strategy	Treatment details	Outcomes & conclusions
CHOICE (Renú et al., 2022)	Anterior circulation LVO; EVT successful recanalization	Adjunct thrombolysis	IA alteplase after thrombectomy	Improved 90-day functional outcome; acceptable safety; microcirculation not directly assessed
PEARL (Writing Committee for the PEARL Investigators, 2025)	Anterior circulation LVO; EVT successful recanalization	Adjunct thrombolysis	IA alteplase 0.225 mg/kg post-EVT	Higher mRS 0–1 at 90 days; similar sICH; indirect microvascular benefit
ANGEL-TNK (Miao et al., 2025)	Anterior circulation LVO; incomplete recanalization (eTICI 2b)	Adjunct thrombolysis	IA tenecteplase after EVT	Improved excellent functional outcome in selected patients; no excess sICH; no direct microcirculation endpoint
POST-TNK (Huang et al., 2025)	Anterior circulation LVO; near-complete recanalization (eTICI 2c–3)	Adjunct thrombolysis	IA tenecteplase post-EVT	No functional benefit; increased any ICH; limited value when reperfusion is optimal
POST-UK (Liu et al., 2025)	Anterior circulation LVO; successful EVT	Adjunct thrombolysis	IA urokinase after EVT	Neutral functional outcome; higher hemorrhagic risk; microcirculation not evaluated
ATTENTION-IA (Hu et al., 2025)	Posterior circulation LVO; EVT	Adjunct thrombolysis	IA tenecteplase post-EVT	No significant functional benefit; safety acceptable; no microcirculation data
Tirofiban RCT (Zhao et al., 2024)	Non-cardioembolic AIS; small–medium vessel	Antiplatelet	IV tirofiban vs. aspirin within 24 h	Reduced early neurological deterioration; no increase in sICH
ASSET-IT (Tao et al., 2025)	Non-cardioembolic AIS; no EVT	Antiplatelet	IV tirofiban within 60 min after IVT; 24 h infusion	Improved 90-day functional independence; low sICH; microcirculation not assessed
Tirofiban + EVT, a PSMA (Guo W. et al., 2022)	AIS; successful recanalization (mTICI 2b or 3)	Antiplatelet	IV or IV + IA tirofiban	IA + IV tirofiban improved mRS and reduced mortality at 90days; no increase in ICH or sICH
ApTOLL + EVT (Hernández-Jiménez et al., 2023)	Anterior circulation LVO; EVT	Anti-inflammatory (TLR4 inhibition)	IV ApTOLL peri-EVT	Reduced mortality and disability at 90 days; microcirculation not directly measured
MARVEL (MARVEL Trial Authors for the MARVEL Investigators, 2024)	AIS; EVT	Anti-inflammation and anti-cerebral edema	IV methylprednisolone 2 mg/kg/d ×3 days, first <2 h after arterial access closure	No significant difference in mRS 90 days; lower mortality rate; lower sICH
TASTE-2 (Wang et al., 2026)	Anterior circulation LVO; EVT planned	Anti-inflammatory, antioxidant	Edaravone–dexborneol before thrombectomy	Higher 90-day functional independence; safe; no direct microcirculation endpoint
CRYSTAL (Wang, 2023a)	AIS; anterior circulation LVO; EVT with successful recanalization	Antiplatelet + anti-inflammatory	Cilostazol + dexborneol (Y-6); start ≤2 h post-reperfusion; 28 days	primary mRS at 90 days; microcirculation dysfunction assessed; **On-going**
Cerebrolysin + EVT (Eichel, 2023)	AIS; hemispheric stroke; EVT	Neurovascular protection	Cerebrolysin 30 mL IV daily ×10 days; start ≤3 h post-EVT	mRS 90 days; CT-perfusion outcomes; **On-going**
ESPRIT (Wang, 2023b)	AIS; reperfusion therapy	BBB protection	Sarecycline Tablet,100 mg daily ×7 days; start <30min after randomization	Changes of NIHSS score at 7 days; Venous thrombotic inflammation indicators; **On-going**
Fingolimod + EVT (Second Affiliated Hospi tal et al., 2023)	AIS; bridging therapy (IVT + EVT)	Immune modulation	Fingolimod 0.5 mg PO daily×3 days; start about 1 h before EVT	mRS 90 days; infarct volume; **On-going**
IRIS (Suzhou Municipal Hospital of Anhui Province, 2024)	AIS; anterior circulation LVO; EVT	Targeted anti-inflammatory (IL-6R inhibition)	Single-dose IV tocilizumab peri-EVT	infarct volume at 72 h; Completed, results unpublished
IRIS-2 (Xunming and PhD, 2025)	AIS; anterior circulation LVO; EVT	Targeted anti-inflammatory (IL-6R inhibition)	IV tocilizumab peri-EVT vs. placebo	safety and efficacy; **On-going**

Abbreviations: AIS, acute ischemic stroke; eTICI, expanded Thrombolysis in Cerebral Infarction; EVT, endovascular thrombectomy; ICH, intracerebral hemorrhage; IVT, intravenous thrombolysis; IV, intravenous; IA, intra-arterial; LVO, large-vessel occlusion; mRS, modified Rankin Scale; NIHSS, national institute of health stroke scale; PO, oral administration; PSMA, propensity score matching analysis; sICH, symptomatic Intracerebral hemorrhage.

One clinically consequential “macro-to-micro” issue is blood pressure management after the procedure. Randomized studies suggest that intensive blood pressure lowering after successful recanalization does not improve outcomes and may even worsen early neurological status (Mazighi et al., 2021; Yang P. et al., 2022), plausibly because it further stresses a vulnerable downstream microcirculation already challenged by oxidative stress, inflammation, and capillary flow limitation.

#### 5.2.2 Adjunct intra-arterial thrombolysis

Adjunct intra-arterial thrombolysis following thrombectomy may represent one of the few approaches with repeated clinical signals compatible with improved tissue-level reperfusion. Both CHOICE and PEARL reported better 90-day functional outcomes without an excess of symptomatic intracranial hemorrhage (Renú et al., 2022; Writing Committee for the PEARL Investigators, 2025). Mechanistically, the rationale is straightforward: distal microthrombi/microemboli can perpetuate hypoxia even after large-vessel reopening, thereby fueling infarct expansion, endothelial/BBB injury, edema, and hemorrhagic transformation (Ji et al., 2023; Singer et al., 2025; Mujanovic et al., 2023; Mujanovic et al., 2025b). The major limitation is interpretive: most trials did not include microcirculation-specific endpoints (e.g., angiographic microcirculation time or capillary-phase delay; residual hypoperfusion volume), so mediation by true microvascular reopening is often assumed rather than demonstrated.

By contrast, larger trials such as POST-TNK, POST-UK, and ATTENTION-IA were neutral (and in some analyses suggested worse bleeding) when adjunct lysis was added after near-complete reperfusion (Hu et al., 2025; Huang et al., 2025; Liu et al., 2025), consistent with a ceiling effect once macroreperfusion is already highly optimized. In ANGEL-TNK, however, benefit was suggested in selected patients with incomplete recanalization (eTICI 2b) and favorable mismatch (Miao et al., 2025), implying that patient selection based on residual downstream impairment may be decisive for whether additional lysis can meaningfully improve microcirculatory reperfusion.

#### 5.2.3 Adjunct antiplatelet therapy

Peri- or post-recanalization glycoprotein IIb/IIIa inhibition, most commonly with tirofiban and less extensively with eptifibatide, has been associated in some studies with improved neurological stability and functional outcome without an obvious increase in hemorrhagic complications (Zhao et al., 2024; Guo W. et al., 2022; Zhao et al., 2017; Tao et al., 2025). These findings fit a model in which platelet activity contributes to microvascular injury. Still, microcirculation-focused imaging endpoints, such as capillary-phase angiographic delay or residual perfusion deficit, are generally not reported, which weakens causal attribution to microvascular reopening.

#### 5.2.4 Adjunct anti-inflammatory/anti-ROS strategies

ApTOLL (a TLR4 antagonist) and methylprednisolone have shown encouraging signals as EVT adjuncts, including reduced disability/mortality or favorable safety profiles (MARVEL Trial Authors for the MARVEL Investigators, 2024; Hernández-Jiménez et al., 2023). Edaravone–dexborneol started before thrombectomy improved functional independence in TASTE-2 (Wang et al., 2026). Yet, in most studies the key endpoints are global injury measures, clinical outcomes, or hemorrhagic risk, rather than direct evidence that microvascular flow was restored.

#### 5.2.5 Ongoing trials

Ongoing trials include Y-6 sublingual tablets (cilostazol + dexborneol) (Wang, 2023a), sarecycline (Eichel, 2023), cerebrolysin (Wang, 2023b), fingolimod (Second Affiliated Hospi tal et al., 2023), and tocilizumab (Xunming and PhD, 2025; Suzhou Municipal Hospital of Anhui Province, 2024), along with further evaluation within SPAN frameworks (Fisher and Savitz, 2022; European Stroke Journal, 2025). Although these agents may affect microcirculation through antiplatelet, anti-inflammatory, neuroprotective, or immunomodulatory mechanisms, most programs still rely primarily on clinical endpoints and conventional imaging; dedicated microcirculatory assessment remains the exception rather than the rule.

Overall, while several adjunctive strategies have shown signals of clinical benefit, the underlying mechanisms remain uncertain, as most studies lack direct microcirculatory endpoints. Observed effects may reflect multiple processes, including collateral status improvement or modulation of tissue injury, rather than capillary-level restoration alone. improved outcomes should not be automatically equated with microcirculation reversal. The field’s central task is therefore not merely to add more candidate drugs, but to connect interventions to rational stratification and to quantifiable measures of residual microcirculatory dysfunction. Integrating angiography-based microcirculation time indices and follow-up perfusion-defined residual hypoperfusion (Ji et al., 2023; Singer et al., 2025; Mujanovic et al., 2023; Mujanovic et al., 2025b) will be essential for identifying true no-reflow and for selecting patients most likely to benefit from mechanism-matched adjunct therapy.

### 5.3 Why robust preclinical efficacy often fails to translate in patients

Despite strong mechanistic plausibility and reproducible efficacy in experimental models, many attempts to target post-stroke microcirculatory dysfunction yield neutral or heterogeneous clinical results, reflecting a recurring translational failure (Turner and Farr, 2024). Several barriers contribute to this gap.

Species differences represent a fundamental constraint: compared with humans, rodents differ in cerebrovascular architecture, collateral organization, immune responses, and neurovascular unit composition, limiting how directly mechanistic insights can be generalized (Narayan et al., 2021). Timing mismatch is another major issue. In experimental studies, therapies are commonly administered before or immediately after recanalization while platelet aggregation, leukocyte adhesion, oxidative stress, and pericyte contraction are still evolving, whereas in clinical practice adjunct treatments often begin only after successful thrombectomy, at a point when microvascular obstruction may already be structurally established (Turner and Farr, 2024; Mujanovic et al., 2024). In addition, human ischemic stroke is deeply heterogeneous in age distribution, comorbidity burden, infarct core size, collateral status, clot composition, and reperfusion quality, which can dilute effects that appear strong in standardized preclinical cohorts (Singh et al., 2022).

Most importantly, microcirculatory failure is rarely driven by a single, isolated pathway. Instead, it reflects a convergence of microthrombosis, endothelial dysfunction, inflammatory and oxidative injury, cellular stalling, and pericyte-mediated capillary constriction (Sun et al., 2015). Consequently, broadly acting therapies, often vasodilatory or antithrombotic agents with overlapping anti-inflammatory/antioxidant effects, are unlikely to succeed consistently as monotherapies unless they are aligned with the dominant mechanism in carefully selected patient subgroups (Blase et al., 2025). Together, these factors help explain why interventions with compelling mechanistic narratives do not reliably translate into consistent clinical benefit. Future research should integrate more clinically relevant experimental designs and human-relevant platforms, such as stem cell–derived brain and vascularized organoids (Kistemaker et al., 2025; Wang et al., 2023), to complement animal models. And an integrated approach may accelerate the development of adjunctive therapies targeting the cerebral microcirculation and improve functional recovery after ischemic stroke.

## 6 Summary and perspective

This review identifies post-recanalization microcirculatory dysfunction as a clinically relevant and biologically plausible contributor linking successful macrovascular reopening with incomplete tissue reperfusion and poor neurological recovery. However, its relative importance remains uncertain, as current evidence does not permit clear prioritization over other established contributors to FR, including collateral status, ischemia duration, and baseline tissue injury. Accordingly, microcirculatory-targeted strategies are most relevant in patients with remaining salvageable tissue, rather than in those with already completed infarction.

Microcirculatory dysfunction is both downstream and self-propagating: ischemia/reperfusion processes (oxidative stress, inflammation, endothelial injury, microthrombosis, and pericyte dysfunction) initiate the damage, but the resulting microvascular compromise can become self-sustaining and drive persistent hypoperfusion and secondary injury. Across microvascular compartments, capillary-level failure, through microthrombotic plugging, immune-cell stalling, and pericyte-mediated constriction, appears to be a recurrent and potentially important determinant of impaired microcirculatory reperfusion. Parallel advances in vivo assessment, from high-resolution optical imaging in experimental models to angiography and perfusion based clinical metrics, reinforce a key interpretive point: restored blood volume does not necessarily imply restored blood flow. The growing capacity to distinguish capillary perfusion, flow velocity, and functional capillary density clarifies why macrovascular success is an insufficient surrogate endpoint and why microcirculatory assessment should be incorporated into mechanistic interpretation and therapeutic evaluation.

Future progress is likely to depend less on expanding the list of candidate drugs and more on matching mechanism-specific interventions to timely, clinically feasible microcirculatory readouts. Strategies that directly address capillary obstruction biology, particularly neutrophil-platelet interactions, microthrombosis, endothelial glycocalyx injury, and persistent pericyte constriction, may be most effective within a narrow peri-recanalization window that extends into the early hours after reperfusion, before obstruction becomes structurally entrenched. An important remaining gap is the lack of standardized, validated tools to detect residual microcirculatory failure at the bedside. Such tools would strengthen mechanistic inference, enable patient stratification, and support imaging-guided precision trials. Harmonizing angiography-derived microvascular perfusion measures with perfusion imaging–defined hypoperfusion, and shifting clinical endpoints toward capillary-level reperfusion rather than vessel patency alone, will be essential. Experimental and translational data showing neutrophil driven capillary stalling (El et al., 2020) and persistent pericyte constriction (Shrouder et al., 2024) after recanalization provide a strong biological foundation for this direction. Ultimately, integrating microcirculation detection, timing, and mechanism-matched therapy offers a realistic path to narrowing the lasting gap between experimental success and clinical efficacy in ischemic stroke.
